# Research on the Degradation and Failure Mechanisms of the Unclamped-Inductive-Switching Characteristics of p-GaN HEMT Devices

**DOI:** 10.3390/mi16050514

**Published:** 2025-04-27

**Authors:** Li Liu, Yulu Zhen, Siqiao Li, Bo Pang, Kai Zeng

**Affiliations:** 1 State Key Laboratory of Wide-Bandgap Semiconductor Devices and Integrated Technology, School of Microelectronics, Xidian University, Xi’an 710071, China; 2 Guangzhou Institute of Technology, Xidian University, Guangzhou 510555, China; zhenyulu9245@163.com (Y.Z.); leesq123@163.com (S.L.); pb2147705204@163.com (B.P.); zengkk1012@163.com (K.Z.)

**Keywords:** p-GaN HEMT, UIS, failure analysis, degradation and recovery

## Abstract

Single UIS and repetitive UIS experiments are performed in this article to expound physical failure mechanisms in P-GaN HEMT devices. V_peak_ and I_peak_ are used as metrics to evaluate the degradation of electrical parameters. In the single UIS tests, different load inductors, off-gate voltages, and ambient temperatures are chosen as variables to observe the failure phenomena in the device under test (DUT), while in the repeated UIS tests, the threshold voltage, on-state resistance, blocking characteristics, and gate leakage current degradation and recovery are analyzed, and it is concluded that Vth presents a negative shift, R_on_ and BV are restored to their initial value, and gate leakage shows a significant reduction at first and then, after a duration of lagging, gradually recovers to some extent, but is unable to achieve its initial value. Combining failure point analysis via decapping with TCAD simulation and validation, it is found that hole trapping and detrapping in the p-GaN region dominate Vth and Igss degradation, while electron traps in the buffer dominate R_on_ and BV degradation.

## 1. Introduction

Due to their excellent electronic properties, such as their high electron saturation velocity and high breakdown voltage, GaN-based high-electron-mobility transistors (HEMTs) have been confirmed to be a leading transistor technology for future high-power devices during high-frequency operations. However, one of the critical disadvantages of GaN power devices is the lack of unclamped-inductive-switching (UIS) capabilities [1,2]. It is well acknowledged that due to the existence of inductive loads and parasitic components in circuits, power devices may also suffer from UIS strikes, which occur during the switch-off process. Theoretically, if the inductors are unclamped, the current stored in the inductors will surge through the power device from the drain to the source during the switch-off transient, and then, the voltage drop on the device will increase rapidly. In this way, the device suffers from high-current and high-voltage stresses at the same time, which may lead to severe parameter degradations.

As is known, because of the lack of an avalanche process in GaN HEMT devices, they possess weaker UIS capabilities compared to their Si-/SiC-based counterparts. For Si-/SiC-based devices, avalanche energy (i.e., the surge energy during the UIS process) is the decisive factor in damaging the devices. The avalanche is an impact ionization (I.I.) and multiplication process that usually occurs at the p-n junction. There are two different views about UIS failure in P-type GaN HEMTs. One is related to avalanche multiplication, and the other is not avalanche-related, instead focusing on LC resonant circuits. Device failure is only related to the high electric field under high voltage [3].

Bao et al. [4] and Sheng Li et al. [5] think that avalanche breakdown can be found in P-type GaN HEMT devices to a certain extent, while the UIS avalanche energy they can withstand is relatively small, at only the µJ level. The holes generated by impact ionization will accumulate and give rise to avalanche multiplication and current increase, leading to thermal runaway. Naka and Saito reported that the UIS withstanding capability of p-GaN HEMTs can be designed from the viewpoint of hole removal, which is related to the gate leakage current. Marek et al. analyzed the influences of different load inductances and different supply voltages on the UIS withstanding capability of p-GaN HEMTs. Reference [6] presented the results of repetitive UIS stress on normally on HEMTs; moreover, a deep-level transient spectra (DLTS) analysis was taken to study the degradations of off-state leakage current to determine whether holes or electrons are the dominant factor of device failure. Many researchers think that avalanche phenomena and the charge captured by defects exert a profound influence on GaN devices, and the capture mechanisms of defects between the interface of the heterojunction and the barrier are attributed to the cause of electrical performance under UIS stress [7,8,9]. In 2022, Zhang et al. [10] clarified the surge-energy withstanding mechanisms and failure physics of commercial GaN GITs. They summarized their main differences compared to those of Si and SiC MOSFETs. Their results suggested that the avalanche energy, a widely used JEDEC standard for the robustness of Si and SiC power MOSFETs, which represents the device capability to resistively dissipate energy without thermal runaway, may not be an appropriate parameter that can directly represent the surge-energy robustness of GaN HEMTs. Avalanche robustness in GaN HD-GITs depends on the overvoltage pulse created by the UIS test, and additional FIB analyses show that the failure point was near the drain region. Simultaneously, they thought that the variation in V_peak_ versus I_peak_ could preferably evaluate the UIS capabilities of GaN devices.

To clarify the electrical failure behavior of p-GaN HEMTs under UIS stress, single and repetitive UIS stress measurements were performed with V_peak_ and I_peak_ chosen as the criteria of parameter degradation. In this article, commercial products from GaN System Inc. (GS66508T), which can withstand high voltages above 650 V and conduct high currents above 30 A, are adopted as the studied target devices. The influence of different load inductors, V_gs_off_, and ambient temperature on the characteristics of the DUT under a single UIS pulse is measured and evaluated. Meanwhile, under repetitive UIS stress, the threshold voltage, on-state resistance, blocking characteristics, and gate leakage degradations and recovery mechanisms are elucidated. Finally, a combination of decapsulation and failure location analysis and TCAD simulation is used to systematically and comprehensively explore the failure mechanisms of the DUT under UIS stress.

## 2. Device Structure and Experimental Setup

**Figure 1:**
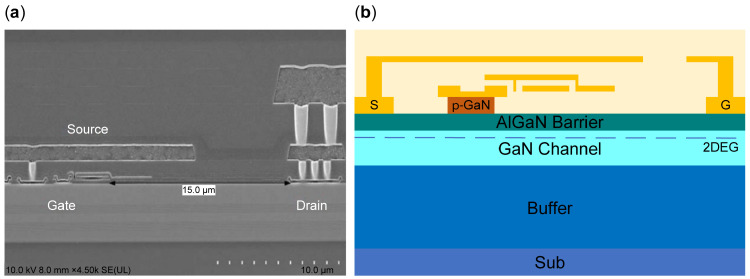
Cross-section of the p-GaN HEMT: (**a**) SEM image from [11], (**b**) illustrative drawing of the DUT used in this work.

Figure 1 gives the cross-section of the p-GaN HEMT used in our work. The measurements were taken using DUTs listed as GaN Systems GS66508T, as shown in Figure 1a, as an enhancement-mode Schottky P-Gate GaN-on-silicon HEMT rated at 1.1~2.6 V, 30 A, 650 V, and 50 mΩ with an area of 6.9 mm × 4.5 mm encapsulated in surface-mounted packages with top-side cooling. Figure 1b provides an illustrative drawing of the DUT in our work.

**Figure 2:**
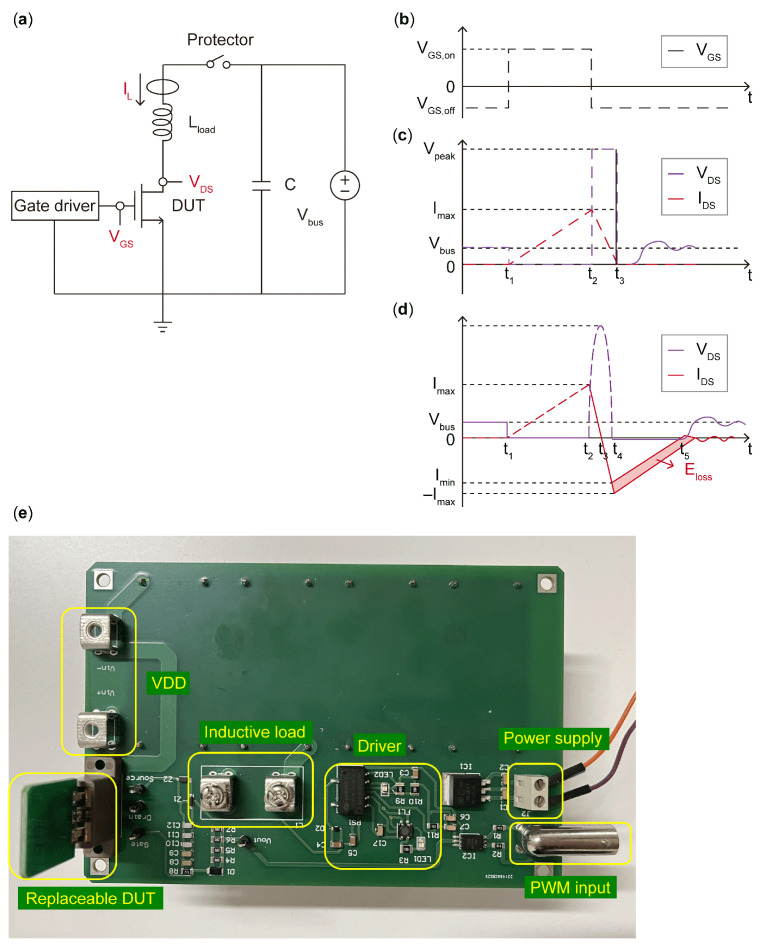
Topology of the UIS test circuit and test board, (**a**) UIS test circuit, (**b**) V_GS_ waveform, (**c**) V_DS_ and I_DS_ under UIS stress of the Si/SiC MOS, (**d**) V_DS_ and I_DS_ under UIS stress of the GaN HEMT, and (**e**) the test board.

Figure 2a shows a typical UIS test schematic; Figure 2b shows a typical gate pulse V_GS_ applied to the gate electrode of the DUT; Figure 2c,d show V_DS_ and I_DS_ waveforms under UIS stress of the Si/SiC MOS and the GaN HEMT, respectively; and Figure 2e presents the back of the test board. The VDD, inductive load, DUT, gate driver, and PWM input are marked with a yellow frame. Different from Si/SiC devices with dynamic UIS avalanche processing, it is well known that there is no traditional pn junction connecting the source and the drain of GaN HEMTs, which adhere to current transport theory due to heterojunction and 2D electron gas channel existence, leading to a minimal avalanche capability and even no avalanche capability. Comparing the UIS waveforms of GaN HEMTs with the ones of Si/SiC MOSFETs in Figure 2c,d, when V_GS_ switches off, the energy stored in the load inductor flows through the DUT; meanwhile, because of the lack of avalanche process, the V_DS_ applied to the GaN HEMT increases gradually to the maximum value, while, comparatively, the voltage dropped between the drain and the source of the Si/SiC MOSFET is clamped to a constant value. As the displacement current appears, the V_DS_ drop on the GaN HEMT goes down. The entire UIS period seems to be an LC resonant circuit. Notably, when the loop current I_DS_ in Figure 2d drops to zero, simultaneously, V_DS_ achieves a peak value. And after that, I_DS_ decreases further to a negative value. The current value at t4 equals to the one at t2 and this phenomena indicates that the dissipation of the energy stored in a load inductor does not rely on avalanche processes, and instead relies on the LC resonant circuit of the load inductor and parasitic capacitance in the body and thus may lead to severe parameter degradations and even catastrophic failure [10].

Actually, due to measurement errors and other uncontrollable factors, the zero point of the current in Figure 2d is usually not coincident with the time point of the maximum value of the voltage; that is, t3 will shift a little. During the t3~t4 period, energy stored in the output capacitance, Coss, of the DUT will dissipate through the load inductor; since the existence of Ron and charge imbalance is caused by the trapping and detrapping of the dynamic R_on_ [12] of the DUT, energy consumption Eloss occurs but only at uJ level. Surge energy cannot be dissipated through avalanche processes as observed in Si/SiC counterparts and could instead be consumed by LC resonance, consisting of the load inductor and Coss [13]. When t4 is reached, V_DS_ generated by LC resonance can induce a negative value and exceed its reverse threshold voltage; thus, the DUT is in the third working operation [14]. At this moment, the circuit will dissipate the energy through the power supply of the VDD loop [15,16].

## 3. Parameter Degradation and UIS Ruggedness Analysis

### 3.1. Single UIS Test

**Figure 3:**
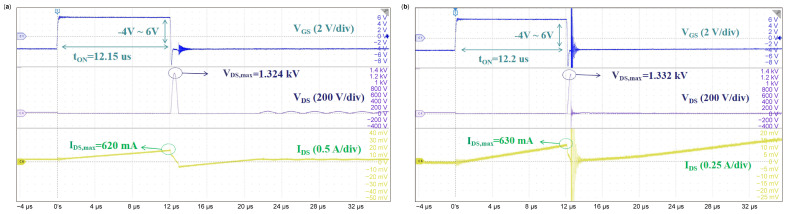
Typical single-pulse UIS waveforms with a load inductor of 0.3 mH at (**a**) the last test before failure and (**b**) at failure.

Figure 3 shows the typical single UIS waveforms of p-gate GaN HEMTs at room temperature under 0.3 mH of inductive load conditions; the last waveforms before failure are shown in Figure 3a, while the waveforms at failure are shown in Figure 3b. The test conditions are as follows: the power supply is 30 V, and the gate driver voltage is switched from −4 V to 6 V. From Figure 3a, when a gate pulse width of 12.15 μs is applied on the gate–source of the DUT, the peak current value of the drain–source is 620 mA; when the V_GS_ switches from 6 V to −4 V, the induced voltage is applied between the drain and source with a peak value of about 1.324kV, and at this moment, the current equals to zero. During the entire UIS process, V_DS_ rises from 0 to V_DS_, increases to the max, and then drops to 0; I_DS_ linearly decreases to a negative value, and then increases to 0. The entire process lasts about 0.85 μs and is similar to LC resonance [17]. During the reverse of the I_DS_ period, after a duration of reverse conduction, V_DS_ starts to oscillate and is then dampened to 30 V. In Figure 3b, when the gate pulse width increases to 12.20 μs, the charging period of the inductor increases, and the peak value of I_DS_ reaches 630 mA. Then, after the DUT is switched off, V_DS_ increases rapidly to the highest point of voltage of 1.332 kV, at which the DUT fails. After the DUT fails, V_GS_ remains constant at −4 V, and V_DS_ approaches zero. After this, the current rapidly increases, which shows that the short circuit of the source and drain happens after the DUT fails. At this moment, the DUT behaves like a passive low impedance.

**Figure 4:**
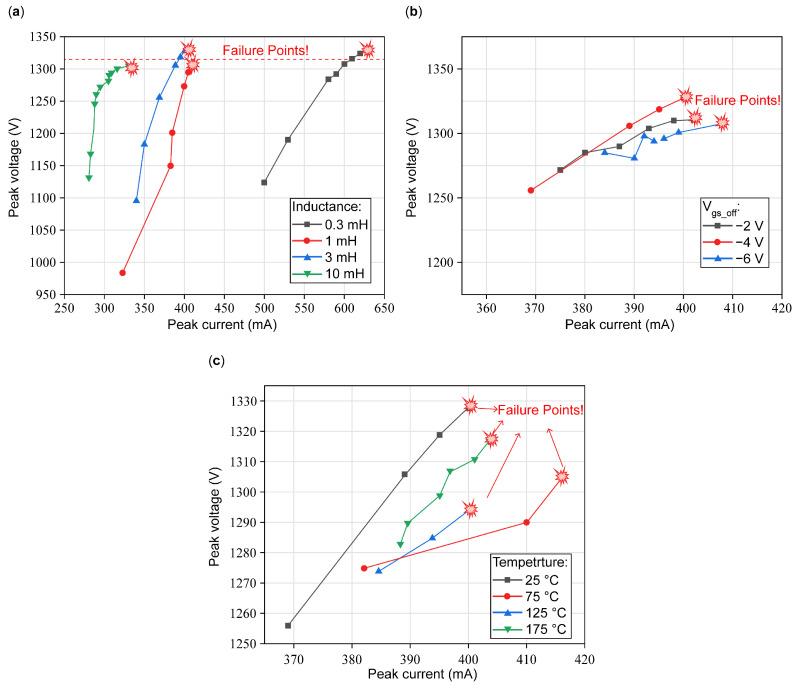
Peak voltage versus peak current during a single UIS test at different (**a**) inductance, (**b**) V_gs_off_, and (**c**) temperature values.

By varying different test conditions such as load inductance, Vgs_off, and temperature, a series of single-pulse UIS tests are conducted by gradually increasing the gate pulse to obtain the failure point, and then, peak currents and peak voltages under different working conditions before device failure are extracted and analyzed, as shown in Figure 4. Data points in Figure 4a are obtained under different inductance, (Figure 4b) V_gs_off_, and (Figure 4c) temperature values. In Figure 4, a total of nine chips are used to obtain the data points. Every point in each curve in these three plots is extracted by increasing the gate pulse width until the chip reaches the failure state marked with a red explosion.

It can be seen that in Figure 4a, with inductance increasing, the peak current will obviously decrease because of the increase in the charging and discharging period of the load inductance, and the UIS process period will also increase. Meanwhile, critical V_DS_ shows little floating before the device failed and a less significant relationship with load inductance. In Figure 4b, we can see that both the peak current and the critical peak voltage between the drain and source show a little floating, which may be caused by device differences, and have no obvious changes with the variation in V_gs_off_. During the reverse-bias p-GaN/AlGaN/GaN diode, the drain-to-gate voltage drop remains, and the barrier height of the Schottky-metal/p-GaN junction can be neglected. As a result, V_gs_off_ has little influence on the UIS withstanding capability of the DUT. Figure 4c presents critical voltage versus peak current before failure under different ambient temperatures, shows no obvious relationship with temperature, and looks more likely to have some fluctuations. As investigated in [18], the Coss changes slightly; therefore, the V_peak_ cannot be influenced by temperature. In this study, our experimental results have a similar tendency to [14,18], which verifies our workbench platform and method. Differences in numerical values come from various uncontrolled factors and X- and Y-coordinate axis range settings.

**Figure 5:**
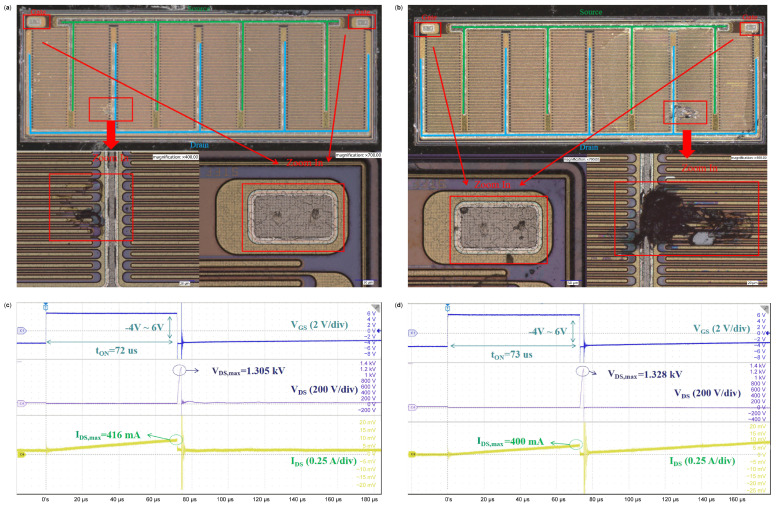
Decapped failure samples and captured curves at failure: (**a**,**b**) decapped failure point analyses of the DUT; (**c**) measured UIS waveforms when (**a**) failure occurs: T = 75 °C, V_GS_ = −4~6 V, L = 3 mH, VDD = 30 V; (**d**) measured UIS waveforms when (**b**) failure occurs: T = 25 °C, V_GS_ = −4~6 V, L = 3 mH, VDD = 30 V.

In order to verify the failure mode under a single UIS test, the failed sample with a different failure mode is decapped and delayered; after local corrosion treatment without damage, an optical microscope (OM) is used to observe and validate the failure point by adjusting magnification. Figure 5 shows the OM images of decapped failure samples and waveforms at failure. Figure 5a,b show the decapped results of the failed devices. Figure 5c,d are measured UIS waveforms when (a) and (b) failure occurs, respectively. The failure test is performed under conditions of 75 °C, with V_GS_ switching from −4 V to 6 V, and a load inductance L equal to 3 mH in Figure 5a,c, While a VDD of 30 V and 25 °C, with −4 V~6 V, 3 mH, and 30 V in Figure 5b and Figure 5d, respectively. We can see from Figure 5a that a slight burnout compared with Figure 5b happens in the drain metal strip region and the gate region. We can confirm that the failure is caused by the breakdown of and damage to the drain and gate. Since the burnout area is very small and no sharp increase in I_DS_ occurs in captured curves in Figure 5c, we can confirm that no short circuit occurs between the drain and source. Comparatively, as presented in Figure 5b, the large burnout area is found near the drain, with obviously damaged areas on the surface and around the gate region. So, the main failure reason for the chip is related to the short circuit of the drain and source caused by drain failure, and finally, the chip burnout, as shown in Figure 5b, corresponding to the I_DS_, increases linearly, and V_GS_ rises slightly from zero. Meanwhile, the gate electrode is also subjected to great UIS stress and leads to damage, which is consistent with the captured waveform in Figure 5d.

### 3.2. TCAD Simulation Under Single UIS Stress

**Figure 6:**
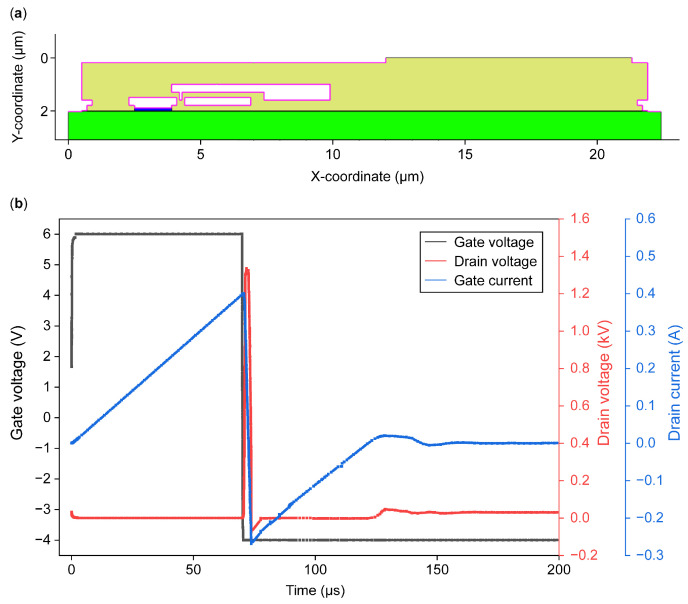
Structure and simulated curves by TCAD: (**a**) cross-section of DUT used in simulation and (**b**) simulated curves by TCAD.

To further explore the UIS failure mechanisms, TCAD simulation is synchronously used to explain the failure behavior. In the simulation, the structure of the p-GaN gate HEMT comprised a 2.0 μm thick GaN layer, a 15 nm AlGaN barrier, a 23% Al layer, and a 100 nm p-GaN layer. Most structure parameters refer to [11,19,20,21], and for calibration, we adjusted some of them. The gate length was 1.5 μm, the source–gate distance was 1 μm, and the gate–drain spacing was 17 μm [19]. The doping concentration of the p-GaN layer was 3 × 10^17^ cm^−3^ [11]. In addition to the above, to coincide well with the experimental data points, some corrections should be carried out in the key structure region. Firstly, interface states are intentionally added to the AlGaN/GaN interface to explain the net polarized charges due to the spontaneous and piezoelectric polarization effects generated in the AlGaN/GaN structure. Furthermore, since body traps play an important role during the UIS processing, acceptor traps in the GaN channel should be introduced, with a concentration of $1\times 10^{16}\;{cm}^{-3}$ and an energy level higher than the valence band at about 0.9 eV. The trap concentration in the buffer should be much higher than the one in the channel region, and the concentration is calibrated as $1\times 10^{18}\;{cm}^{-3}$ [19] with an energy level similar to the one in the channel traps. Besides the above deep-level traps, owing to the existence of the heterojunction interface and processing treatment, various traps could be introduced. Shallow-level traps in the interface should be taken into consideration as well. With regard to the device heterostructure and actual fabrication process, both shallow traps and deep traps should be considered, with the interface state concentration set as within the range of $0\sim 1\times 10^{16}\;{cm}^{-3}$, and the energy level as 0.05~0.8 eV above the valence band [20,21]. The exact numerical value is calibrated for simulation. After the accurate calibration of the device structure, UIS processing is performed using the Mixed-Mode method. The circuit parameters are set as follows: the load inductor L was set as 3 mH, the driving voltage V_GS_ was about −4~6 V, the supplied bus voltage VDD was set as 30 V, the conduction duration ton was set as 70 μs, and the peak voltage and peak current were, respectively, almost 1.3 kV and 400 mA, which are close to experimental data; the simulated waveforms are shown in Figure 6b.

**Figure 7:**
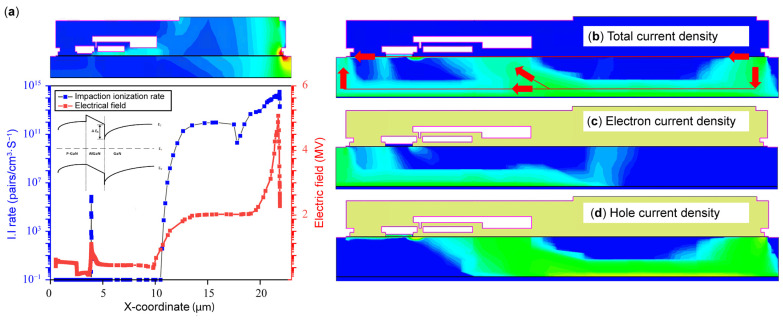
Extracted profiles of DUT at peak voltage (**a**), electrical field and impaction ionization rate (I.I) (**b**), total current density (**c**), electron current density (**d**), and hole current density.

Electrical field and impact ionization versus X-coordinates are extracted at the V_peak_ point, at V_DS_ = 1.3 kV, V_GS_ = 0 V, and 300 K, and plotted in Figure 7a. The energy band of p-GaN/AlGaN/GaN is shown in Figure 7a. The total current density, electron density, and hole density profiles were extracted and are plotted in Figure 7b–d. From Figure 7a, when the DUT suffered from UIS stresses, a huge voltage drop occurred between the source and drain; meanwhile, a large electrical field and strong impact ionization were concentrated around the drain region and then at the edge of the gate field plate and source field plate. We can see from Figure 7b that there exists three current conduction paths: the current from the drain flows through the channel and arrives at the source, the other one is the current from the drain, which passes through the buffer and reaches the source, and the third one is the current that passes through the buffer and flows to the gate. Combining Figure 7a,b, it can be concluded that when the DUT suffers UIS stress increases, a large voltage is generated between the drain and source, and impact ionization will occur under the drain electrode, thereby generating massive electron–hole pairs. Electrons generated are trapped by the buffer while one part of the holes generated move along the channel and pass through the buffer and then move to the substrate; another part of holes move to gate due to the electrical field of the drain to the source and then one part flows into the p-GaN layer and the other part accumulates beneath the gate, ultimately resulting in three current conduction paths. It is concluded from these four figures that DUT failure is mainly caused by a huge electrical field drop at the drain, which exceeds the dielectric limitation, thus leading to dielectric breakdown and damage to the DUT, which is different from the SiC power MOSFET [22,23].

### 3.3. Repetitive UIS Stress

**Figure 8:**
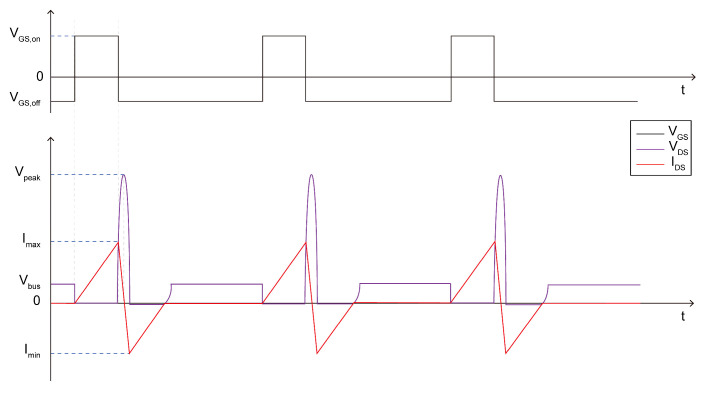
Repetitive avalanche UIS test waveform diagrams.

Figure 8 illustrates the repetitive UIS waveform diagrams. Repeated UIS stress measurements at room temperature are performed in this study, and the test conditions are as follows: the load inductor was equal to 3 mH, the power supply was set to 30 V, the gate bias was switched from −4 V to 6 V, and the conduction duration was 16 us. Under such electrical conditions, each time UIS stress occurs, the V_peak_ between the drain and source reaches 1.1 kV, which is 80 percent of the critical voltage, with I_peak_ equaling 360 mA. Additionally, for effective heat dissipation before the next stress occurs, the duty ratio of the gate pulse is set to 0.1% with a frequency of 62.5 Hz; that is, the interval between each pulse is 16 ms, which ensures that the energy stored in body can be dissipated completely and the influence of temperature can be eliminated. Additionally, a fan is placed close by and used to cool the surface temperature down.

#### 3.3.1. Degradation of Vth

**Figure 9:**
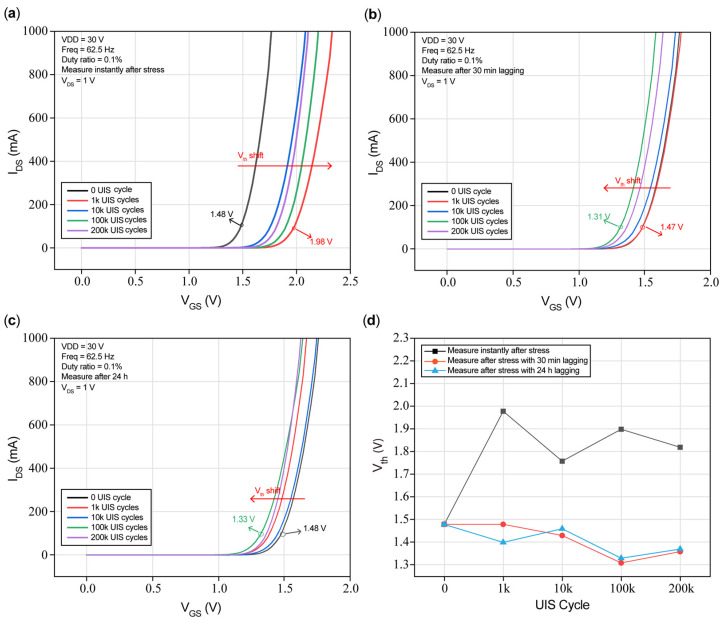
Vth degradation versus cycles: (**a**) measured instantly after cycles, (**b**) measured after 30 min, (**c**) measured after 24 h of lagging, and (**d**) Vth degradation versus cycles.

**Figure 10:**
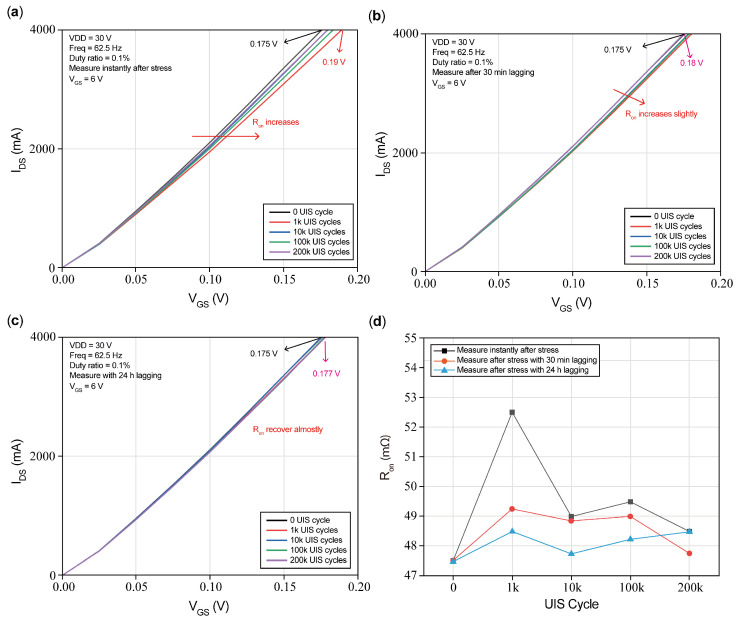
R_on_ versus UIS cycles: (**a**) measured instantly after UIS cycles, (**b**) measured after 30 min of lagging, (**c**) measured after 24 h of lagging, and (**d**) R_on_ versus UIS cycles.

**Figure 11:**
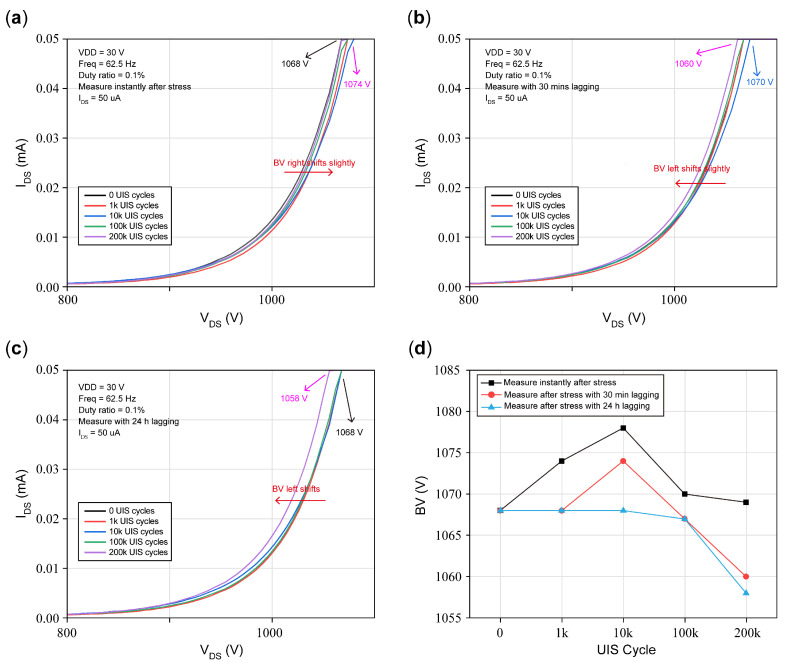
Blocking characteristics under repetitive UIS stresses: (**a**) measured instantly after stresses, (**b**) measured after 30 min of lagging, (**c**) measured after 24 h of lagging, and (**d**) BV versus UIS cycles.

**Figure 12:**
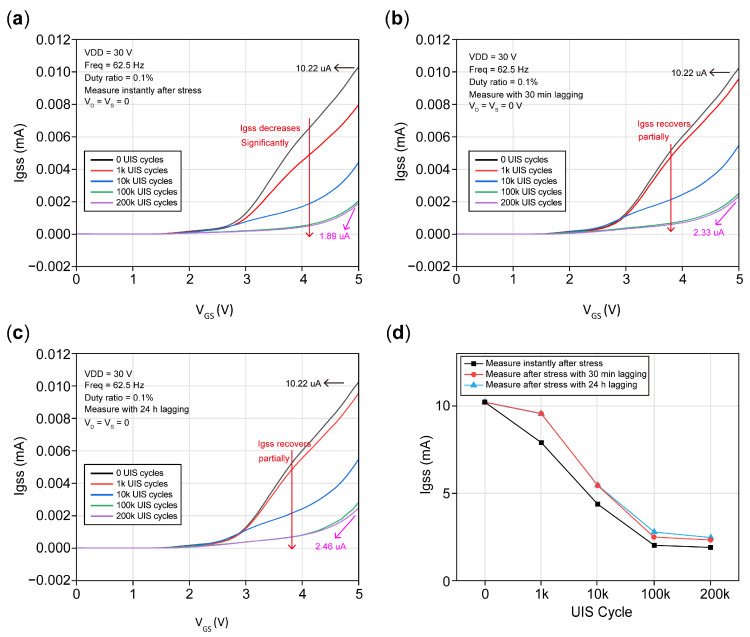
Gate leakage current degradation of GaN HEMTs under repetitive UIS stresses: (**a**) measured instantly after cycles, (**b**) measured after 30 min of lagging, (**c**) measured after 24 h of lagging, and (**d**) Igss versus UIS cycles.

After a certain amount of UIS stress cycles, the following typical static and dynamic electrical performances are measured: transfer characteristic (Vth), output characteristic (R_on_), breakdown voltage (BV), gate-to-source leakage current with short drain-to-source distance (Igss); these are plotted in Figure 9, Figure 10, Figure 11 and Figure 12, respectively.

According to Figure 9, Figure 10, Figure 11 and Figure 12 below, all of the I_DS_~V_GS_, I_DS_~V_DS_, and Igss~V_GS_ plots are captured by a static parameter analyzer. The total number of relevant samples is five. After UIS stress cycles, the static parameter analyzer can evaluate Vth, R_on_, BV, and I_gss_ during one-time testing, and then, after an additional 30 min of lagging and 24 h of lagging, we can capture another set of curves. From Figure 9, Figure 10, Figure 11 and Figure 12, the black curve labeled as zero UIS cycle is adopted as a static electrical reference during the parameter degradation test.

Figure 9 presents measured I_DS_ versus V_GS_ waveforms of the DUT under repetitive UIS stress. The transfer characteristics are obtained from measuring instantly after stress in Figure 9a, with the others being measured after 30 min of lagging in Figure 9b and after 24 h of lagging in Figure 9c. The variation in Vth with cycles measured at I_DS_ = 100 mA is presented in Figure 9d. The test conditions are as follows: VDD = 30 V, Freq = 62.5 Hz, duty ratio = 0.1%, and V_DS_ = 1 V.

Figure 9a shows an obviously positive shifting after 1k, 10k, 100k, and 200k repetitive UIS duty cycles when measuring I_DS_ versus V_GS_ instantly after stresses. That is, threshold voltage will increase; however, there is no reasonable mathematical relationship between shifting level and duty cycles, such as a simple linear relationship proportional to UIS cycles. Generally, the increase in Vth will lead to an increase in R_on_, which is shown in Figure 10a. After the other condition of 30 min of lagging, I_DS_ vs. V_GS_ values were extracted and are plotted in Figure 9b. We found that the Vth will have negative shifts and also that the shifting level has no visible mathematical relationship with UIS cycles. Combining Figure 9a,b, we can conclude that Vth will positively shift at first and then reversely shift after certain UIS cycles, phenomena which are similar to those given in [3,24,25]. And then, after 24 h of continuous lagging, I_DS_ vs. V_GS_ values were extracted and are presented in Figure 9c; the Vth of the DUT continues to exhibit a negative shift compared to when not undergoing UIS stresses, but the shifting degree, to some extent, was reduced compared with the one measured after 30 min of lagging. As illustrated in Section 3.4, the degradation and recovery of Vth and R_on_ are definitely related to the trapping/detrapping of electrons and holes in the gate structure; after the removal of the gate bias, the positive shifts in Vth are explained by electron trapping effects near the gate [3,25], while the negative shifts in Vth after 24 h of lagging is summarized as the trapping of the hole generated by I.I. [26].

In Figure 9d, we can see that after repeated UIS cycles, under different lagging durations, there is no direct linear relationship between Vth degradations during the cycles. Meanwhile, at fixed cycles, Vth shows an obvious reduction. However, with an increase in UIS cycles, the decreasing degree of Vth finally maintains a constant tendency and has a certain degree of reduction compared with the initial value when measuring Vth instantly after stresses. Comparatively, in Figure 9c, after an additional 24 h of lagging, the Vth recovers to around 1.48 V, which has no duty cycles, which presents a recovery tendency, which is similar to [27].

#### 3.3.2. R_on_ Shifts

Figure 10 presents the R_on_ versus UIS cycles with different lagging periods. For simplification, the Y-coordinate axis is set to the same range, and we adopted a variation of V_DS_ as the representative of the R_on_ variation. As described before, the black curve represents the one without UIS stress cycles. Figure 10a presents the I_DS_ versus V_DS_ measured instantly after UIS cycles. An obvious increase in R_on_ is found, and no visible mathematical relationship with repetitive UIS cycles is seen. The DUT undergoing 1k UIS cycles achieved maximum R_on_ increase, and then so did the one subjected to 100k UIS cycles. I_DS_ versus V_DS_ is measured again after an additional 30 min of lagging, and the results are plotted in Figure 10b. From the graph, it is considered that the R_on_ of the DUT increases slightly compared with the one undergoing no UIS cycles, which shows that R_on_ recovered to a certain degree. Further output characteristic measurements are performed continuously after 24 h of lagging and are plotted in Figure 10c. As shown in Figure 10c, after a long period, the R_on_ of the DUT almost recovered to its initial state, while a small increase was found when the DUT underwent 200k UIS cycles. Generally, the increase in and recovery of R_on_ result from the trapping/detrapping of electrons in the buffer layer and carriers stored in the p-GaN layer [27], which is consistent with the physics mechanism of R_on_ shifting. Besides this, it is obviously indicated in Figure 9 and Figure 10 that the increase in R_on_ here is relatively small compared with the Vth shift, which can be attributed to some damage near the gate region [3].

The variation in R_on_ versus UIS cycles is calculated from output characteristics and plotted in Figure 10d. The working conditions are as follows: VDD = 30 V, Freq = 62.5 Hz, duty ratio = 0.1%, and VDD = 30 V with V_GS_ = 6 V. Repetitive UIS stress is set as follows: 0 cycles, 1k cycles, 10k cycles, 100k cycles, and 200k cycles. We found, after computation, that the initial value without UIS cycles is 47.5 mΩ; after 1k cycles of stress, when measuring instantly, R_on_ has the maximum increase of 5 mΩ, which is about a 10.5% increase, and reaches 52.5 mΩ. Then, after 30 min of lagging, R_on_ is reduced to 49.25 mΩ with a reduction of −3.25 mΩ and a −6.2% amplitude reduction. It seems that after repeated UIS cycles, R_on_ is finally restored to its initial value, although there exist alternating increases and decreases during the entire UIS cycle, and the shifting tendency is consistent with [28,29].

#### 3.3.3. BV Shifts

Figure 11 presents blocking characteristics under repetitive UIS cycles, and the test conditions are illustrated in the figure. Figure 11a is measured instantly after UIS cycles, which can be found in Figure 11a, showing that the breakdown point shifts to the right, and the shifting degree is not proportional to UIS cycles. Among these five curves, the one that suffered 10k UIS cycles has the maximum rightward shift. After the other 30 min of lagging, the blocking characteristics were measured and are plotted again in Figure 11b. From Figure 11b, all of the breakdown points measured from the DUT move left. And the DUT suffered 200k cycles and mostly shifts left, while the DUT undergoing 10k cycles displays a certain recovery, but compared with the initial state, it shows a slight increase. After the DUT is kept on for 24 h continuously, and its blocking characteristics are measured and then plotted in Figure 11c, showing that almost most curves recovered, and only the curve that suffered 200k cycles shows left shifts.

BV is extracted from I_DS_ versus V_DS_ at I_DS_ = 50 μA with the gate biased at zero, as plotted in Figure 11d. UIS cycle stresses are set as before. The initial BV without UIS cycles is 1068 V. At 10k UIS cycles, most of the BV presents right shifting and reaches 1078 V, about a 0.93 percent increase. After 30 min of lagging, BV was restored to its initial value at 1k UIS cycles; when UIS cycles increased to 200k, BV was reduced to a smaller value than the initial value, and pre-breakdown occurred. After enduring 24 h of lagging, BVs of the DUT suffered 1k, 10k, and 100k cycles, almost remaining constant with the initial value, while BV measured exceeding 200k UIS cycles reduced to 1058 V, a 0.93 percent decrease. Above all, from Figure 11d, we find that with the increase in UIS cycles, if the lagging is the same, the BV increases at first and then drops to the initial value; when UIS cycles applied exceed a certain amount, the BV will decrease gradually and will not return to its original state. But fewer shifts in BV can be attributed to few electrons being trapped and detrapped in the buffer layer [27,30].

#### 3.3.4. Gate Leakage Degradation

Figure 12 shows gate leakage Igss with a short circuit of the drain to source and gradual gate voltage sweeps from 0 to 5 V. The current at V_GS_ equaling 5 V is adopted as Igss. Figure 12a shows the curves measured after instant UIS cycles. It is obvious that gate leakage Igss decreases significantly and is almost proportional to UIS cycles; that is, the more UIS cycles occur, the more Igss decreases. The trapping effect is considered to be the dominant reason for the decreases in Igss [3]. Figure 12b shows the Igss after 30 min of lagging, and it was found that gate leakage Igss increases slightly compared with that in Figure 12a. Igss increases from 1.89 uA to 2.33 uA after suffering from 200k cycles. Meanwhile, the DUT suffered from 1k cycles and almost recovered to its initial value, and the red curve almost overlaps with the black one. After another 24 h of lagging, Igss was measured and is plotted in Figure 12c; we found that the DUT suffering from 1k and 10k cycles of strikes did not recovered, while the DUT suffering from 200k recovers to its inital state after 100k cycles, which is reflected in the fact that the purple curve overlaps with the green one. Therefore, to suppress gate leakage, many methods are adopted. Such as gate structure improvement, and E-mode pn junction/AlGaN/GaN HEMTs [31] can significantly reduce gate leakage and increase the gate driver bias windows [32].

Figure 12d demonstrates the Igss versus UIS cycles. All Igss values were extracted, as shown in Figure 12a–c, with 0 UIS cycles, 1k cycles, 10k cycles, 100k cycles, and 200k cycles under Vg = 5 V. Obviously, it can be found that Igss decreases at first and is linear to UIS cycles, and then reduces to a constant value. The initial Igss is 10.22 uA without any UIS cycle stress; after 200k cycle strikes, and then measuring instantly, Igss was reduced to 8.33 uA with an amplitude decrease reaching 81.5%. In Figure 12d, the red curve almost overlaps with the blue curve, which means that after a long time of lagging, the DUT remains in a stable state but will not return to its initial state, as indicated by the distance from the black curve. Therefore, the DUT with 24 h of lagging remains stable, and Igss reduces to 2.46 uA, a 75.9 percent reduction compared with 7.76 uA measured instantly after cycles. Conclusively, Igss can return to its initial level within a range of UIS cycles, but if the cycles exceed a special value, Igss barely recovers and shows a significant reduction compared with zero UIS cycles, which is indicated in Figure 5. It can be seen from Figure 5 that the gate will suffer some damage under a single UIS stress. Although the repeated UIS stress will not lead to the complete failure of the DUT, it can be speculated that due to certain traps, it will be introduced into the p-GaN layer; the gate of the DUT will also suffer certain irreversible damages under the repeated UIS stress, which will aggravate the trap effect, so Igss can hardly be restored to the initial level after degradation.

### 3.4. Degradation Mechanisms Under Repetitive UIS Stresses

**Figure 13:**
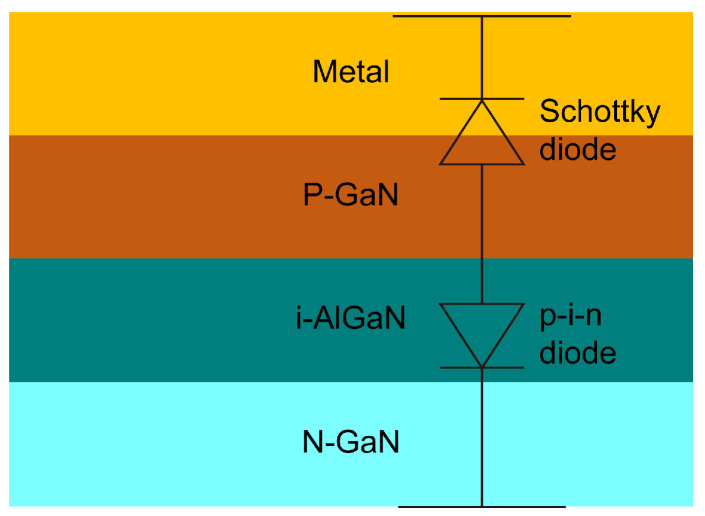
Equivalent circuit of the gate region.

Figure 13 shows the commonly used equivalent circuit model of the gate region in p-GaN HEMTs [3,10,27], which is used to interpret electrical parameter degradation. The gate region is formed by a combination of back-to-back Schottky diodes and p-i-n diodes. Schottky diodes are formed by gate-to-metal junctions, and p-i-n diodes are formed by p-GaN/i-AlGaN/N-GaNs. Due to the bandgap discontinuity in the interface and defects induced by processing, traps cannot be ignored [33,34].

**Figure 14:**
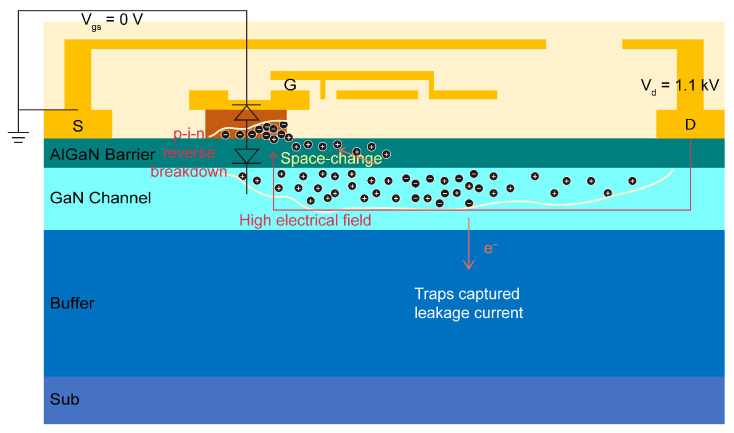
Charge transport when the UIS occurs in p-GaN HEMTs.

Figure 14 illustrates the impact of the ionization process and electrical field profile when UIS stress is applied to the DUT. Because UIS often occurs at the moment V_GS_ switches from Vgs_on to zero, VDD is set to 1.1 kV, and the gate is shorted to the source as shown in Figure 14. When DUT is struck by UIS stress, a large induced voltage is applied between the drain and source. Strong impact ionization will occur in the body, leading to a huge number of electron–hole pairs being generated. A high electrical field will be induced between the drain and gate, marked with a red line; meanwhile, a vertical electrical field directed from N-GaN to p-GaN is generated near the gate electrode, and then induces the p-i-n diode to enter into a reverse blocking state. P-GaN acts as a negative charge center of the space-charge region, while N-GaN acts as a positive charge center of the space-charge region. Holes generated by impact ionization were injected into the gate and barrier and then captured by a trap nearby, while the electrons were captured by traps in the buffer.

When a single UIS is performed as before, the duration of stress only lasts for several microseconds, since electron mobility is much larger than hole mobility. Furthermore, in a short time range after the UIS stress was removed, channel electrons were trapped in electron traps in the GaN buffer, with the amount of holes accumulating beneath the gate. In addition, when the UIS stress is removed, both Schottky diodes and p-i-n diodes are in an off state; there are no extra holes entering the p-GaN region, nor is there recombination with negative space charges, and a negative charge center is left instead. At this moment, to turn on the DUT, a pre-positive voltage should be necessary and applied to the gate electrode to offset the electrical field brought by negative space charges. Thus, threshold voltage consists of two parts: one is the pre-positive gate voltage (△V), and the other is the gate voltage required for channel forming (V_th0_); that is, V_th0_ + △V, in Figure 9a, shows that Vth increases when measured instantly after UIS stressing.

Under the condition of high-voltage bias between the gate and the drain during UIS stresses, electrons become trapped at the interface between the gate and drain which acts as a “virtual gate” that will extend the depletion region beneath the gate to the source and the drain, and additionally exhaust the channel electrons, leading to an increase in R_on_. Since the virtual gate cannot be restored rapidly to its initial state after UIS stresses are removed, when R_on_ is measured instantly after UIS stresses, it will show an increasing tendency. This phenomenon is consistent with that presented in Figure 10a. Besides the effects of the virtual gate, electron traps in the N-GaN buffer also play a role in the I_DS_ decreasing in the off state, which is reflected in Figure 11a, where BV presents an increasing tendency.

As previously described, under the conditions of high gate bias voltage, for a p-i-n diode, a negative charge space is formed in the p-GaN region, and a positive charge space is formed in the N-GaN region. In a short time after UIS stresses are removed, due to both diodes being in an off state, there are no extra holes provided to recombine with negative space charge, and electron traps in the buffer region cannot also immediately release all the trapped electrons to recombine with positive space charges in the N-GaN region. Thus, the existence of space charges will greatly reduce Igss, which is a phenomenon also consistent with that shown in Figure 12a.

After a period where UIS stresses are removed, holes underneath the gate electrode will recombine with the negative space charges in the p-GaN region; thereby, holes will accumulate in the p-GaN region, and Vth will decrease and show negative shifts. Meanwhile, electron traps in the barrier and passivation layer release the trapped electrons, the virtual gate vanishes, and the on-state resistance is gradually restored to its initial value. Electrons released by traps in the buffer will flow into a channel, which results in the number of electrons being larger than holes, and I_DS_ will increase, presented as BV left shifts. Because the space-charge width resolved itself to some extent, gate leakage has also been restored to a certain degree. The above analysis is consistent with the curves with 30 min of lagging in Figure 9b, Figure 10b, Figure 11b and Figure 12b. That is, Vth shows negative shifting, and R_on_ returns nearly to its initial value, while BV barely decreases, and gate leakage with the short circuit of the drain to source is restored to a certain extent.

Under repeated UIS stresses, inside the DUT, continuous impact ionization produces electron–hole pairs, trapped and detrapped electrons, and the recombination of electron–holes, and the space-charge region widens and recovers. During these processes, due to hole mobility being much less than that of electrons, there are barriers above and below the p-GaN layer, making it hard for holes injected into the p-GaN to return to their initial position; when accumulated holes in the p-GaN region in the last stress condition are not consumed not yet, the next stress strikes again. Thus, after repeated UIS stresses, the gate will be damaged permanently, and it is hard for the threshold voltage and gate leakage current to return to their initial levels. However, with the formation and disappearance of a virtual gate, the channel current is dominated by electron traps, and after a long time of lagging, channel resistance and blocking characteristics can almost return to their initial level. However, for the DUT undergoing much more repeated UIS stresses such as 200k strikes, the buffer layer suffers much more, and the blocking characteristics show irreversible degradation.

## 4. Conclusions

Electrical parameter degradations of p-GaN HEMTs under UIS stresses were investigated in this article. Single-pulse and repetitive UIS tests under different working conditions are performed to evaluate parameter degradations and recovery. With the help of TCAD simulation and damage position analysis, we can conclude that the failure mechanism under UIS stress is dominated by dielectric breakdown caused by high electrical fields around the drain electrode and gate damage. However, after UIS stress strikes, Vth, at first, positively shifts and then negatively shifts and finally becomes stable at a negative shifting value, while R_on_ and BV increase at first and gradually return to their initial value. After repetitive UIS striking with different lagging periods, the gate leakage current reduces significantly first and gradually recovers, but cannot completely recover to its initial level. Conclusively, the gate will be damaged by repetitive UIS strikes, and Vth and Igss degradation dominated by hole traps make it difficult to recover to its initial value, while R_on_ and BV degradation dominated by electron traps in the buffer will recover to their initial levels on the whole after a period of lagging.

## Data Availability

The original contributions presented in this study are included in the article. Further inquiries can be directed to the corresponding author(s).

## References

[1] Kozak J.P., Zhang R.Z., Porter M., Song Q.H., Liu J.C., Wang B.X., Wang R., Saito W., Zhang Y.H. (2023). Stability, Reliability, and Robustness of GaN Power Devices: A Review. IEEE Trans. Power Electron..

[2] Ohta H., Asai N., Horikiri F., Narita Y., Yoshida T., Mishima T. (2020). Two-Step Mesa Structure GaN p-n Diodes with Low ON-Resistance, High Breakdown Voltage, and Excellent Avalanche Capabilities. IEEE Electron. Device Lett..

[3] Li S., Liu S.Y., Zhang C., Li N.B., Tao X.Y., Wei J.X., Zhang L., Sun W.F. (2020). Investigations on electrical parameters degradations of p-GaN HEMTs under repetitive UIS stresses. IEEE J. Emerg. Sel. Top. Power Electron..

[4] Bao Q.S., Yang S., Sheng K. UIS withstanding capability of GaN E-HEMTs with schottky and ohmic p-GaN contact. Proceedings of the 2020 32nd International Symposium on Power Semiconductor Devices and ICs (ISPSD).

[5] Kozárik J., Marek J., Jagelka M., Černaj L., Chvála A., Donoval D. Power P-GaN HEMT Under Single and Multi-Pulse UIS Conditions. Proceedings of the 2018 12th International Conference on Advanced Semiconductor Devices and Microsystems (ASDAM).

[6] Marek J., Šatka A., Jagelka M., Chvála A., Príbytný P., Donoval M., Donoval D. Power p-GaN HEMT under unclamped inductive switching conditions. Proceedings of the PCIM Europe 2018; International Exhibition and Conference for Power Electronics, Intelligent Motion, Renewable Energy and Energy Management.

[7] Xie Z.L., Wu X.K., Dong Z.Z., Sun J.H., Sheng K., Chen K.J. (2023). Dynamic On-Resistance Characterization of GaN Power HEMTs Under Forward/Reverse Conduction Using Multigroup Double Pulse Test. IEEE Trans. Power Electron..

[8] Hwang I., Kim J., Chong S., Choi H.S., Hwang S.K., Oh J., Shin J.K., Chung U.I. (2013). Impact of channel hot electrons on current collapse in AlGaN/GaN HEMTs. IEEE Electron. Device Lett..

[9] Vetury R., Zhang N.Q., Keller S., Mishra U.K. (2001). The impact of surface states on the DC and RF characteristics of AlGaN/GaN HFETs. IEEE Trans. Electron. Devices.

[10] Zhang R.Z., Kozak J.P., Xiao M., Liu J.C., Zhang Y.H. (2020). Surge-energy and overvoltage ruggedness of P-gate GaN HEMTs. IEEE Trans. Power Electron..

[11] Chen W.C., Lo H.H., Hsin Y.M. (2024). Threshold Voltage Instability After Double Pulse Test Under Different OFF-State Drain Voltages and ON-State Drain Currents in p-GaN Gate AlGaN/GaN HEMT. ECS J. Solid State Sci. Technol..

[12] Ye J.J., Xuan L., Wu Y.Y., Deng X.C., Li Z.Q., Zhang B. Failure Analysis of 200V p-GaN HEMT under Unclamped Inductive Switching Conditions. Proceedings of the 2021 IEEE Workshop on Wide Bandgap Power Devices and Applications in Asia (WiPDA Asia).

[13] Kozak J.P., Zhang R.Z., Song Q.H., Liu J.C., Saito W., Zhang Y.H. (2021). True breakdown voltage and overvoltage margin of GaN power HEMTs in hard switching. IEEE Electron. Device Lett..

[14] Liu S.Y., Li S., Zhang C., Li N.B., Tao X.Y., Ge C., Qian L., Xin S.X., Sun W.F. (2020). Single pulse unclamped-inductive-switching induced failure and analysis for 650 V p-GaN HEMT. IEEE Trans. Power Electron..

[15] Naka T., Saito W. UIS withstanding capability and mechanism of high voltage GaN-HEMTs. Proceedings of the 28th International Symposium on Power Semiconductor Devices and ICs (ISPSD).

[16] Naka T., Saito W. Relation between UIS withstanding capability and gate leakage currents for high voltage GaN-HEMTs. Proceedings of the 29th International Symposium on Power Semiconductor Devices and IC’s (ISPSD).

[17] Zhang R.Z., Kozak J.P., Liu J.C., Xiao M., Zhang Y.H. Surge energy robustness of GaN gate injection transistors. Proceedings of the IEEE International Reliability Physics Symposium (IRPS).

[18] Li S., Liu S.Y., Tian Y., Zhang C., Wei J.X., Tao X.Y., Li N.B., Zhang L., Sun W.F. (2020). High-temperature electrical performances and physics-based analysis of p-GaN HEMT device. IET Power Electron..

[19] Mixed-Mode Simulation for GaN Power HEMTs in Unclamped Inductive Switching. www.silvaco.com/simulation-standard/TCAD.

[20] Yang W., Yuan J.S., Krishnan B., Shea P. (2019). Characterization of deep and shallow traps in GaN HEMT using multi-frequency C-V measurement and pulse-mode voltage stress. IEEE Trans. Device Mater. Reliab..

[21] Sun R.Z., Lai J.X., Liu C., Chen W.J., Chen Y.Q., Li Z.J., Zhang B. (2021). Analysis of energy loss in GaN E-mode devices under UIS stresses. IEEE Trans. Power Electron..

[22] Scognamillo C., Catalano A.P., Codecasa L., Castellazzi A., d’Alessandro V. (2025). A study of UIS ruggedness of mismatched paralleled SiC MOSFETs. Microelectron. Reliab..

[23] Wang Y.F., Xiao M., Yang Z.N., Porter M., Cheng K., Song Q.H., Kravchenko I., Zhang Y.H. (2025). Robust Avalanche (1.5 kV, 2 kA/cm²) in Vertical GaN Diodes on Patterned Sapphire Substrate. IEEE Electron. Device Lett..

[24] Marek J., Stuchlíková L., Jagelka M., Chvála A., Príbytný P., Donoval M., Donoval D. Impact of repetitive UIS on modern GaN power devices. Proceedings of the 11th International Conference on Advanced Semiconductor Devices & Microsystems (ASDAM).

[25] Xu X.B., Li B., Chen Y.Q., Wu Z.H., He Z.Y., En Y.F., Huang Y. (2020). Investigations on electrical parameters degradation and recovery of E-mode GaN high-electron mobility transistors under repetitive unclamped inductive switching stresses based on low-frequency noise. Semicond. Sci. Technol..

[26] Kozak J.P., Song Q.H., Zhang R.Z., Ma Y.W., Liu J.C., Li Q., Sarito W., Zhang Y.H. (2022). Degradation and recovery of GaN HEMTs in overvoltage hard switching near breakdown voltage. IEEE Trans. Power Electron..

[27] Li S., Liu S.Y., Zhang C., Qian L., Xin S.X., Ge C., Sun W.F. (2022). Comparison Investigations on Unclamped-Inductive-Switching Behaviors of Power GaN Switching Devices. IEEE Trans. Ind. Electron..

[28] Liu C., Chen X.H., Sun R.Z., Lai J.X., Chen W.J., Xin Y.J., Wang F.Z., Wang X.M., Li Z.J., Zhang B. (2023). On the Abnormal Reduction and Recovery of Dynamic R ON Under UIS Stress in Schottky p-GaN Gate HEMTs. IEEE Trans. Power Electron..

[29] Yu R.Z., Jahdi S., Mello P. Performance Instability of 650 V p-GaN Gate HEMT Device under Temperature-related Positive Gate Bias Stresses. Proceedings of the International Exhibition and Conference for Power Electronics, Intelligent Motion, Renewable Energy and Energy Management.

[30] Moens P., Banerjee A., Uren M., Meneghini M., Karboyan S., Chatterjee I., Vanmeerbeek P., Cäsar M., Liu C., Salih A. Impact of buffer leakage on intrinsic reliability of 650V AlGaN/GaN HEMTs. Proceedings of the IEEE International Electron Devices Meeting (IEDM).

[31] Hua M.Y., Chen J.T., Wang C.C., Liu L., Li L.L., Zhao J.L., Jiang Z.H., Wei J., Zhang L., Zheng Z.Y. (2020). E-Mode p-n Junction/AlGaN/GaN (PNJ) HEMTs. IEEE Electron. Device Lett..

[32] Hua M.Y., Wang C.C., Chen J.T., Zhao J.L., Yang S., Zhang L., Zheng Z.Y., Wei J., Chen K.J. (2021). Gate Current Transport in Enhancement-Mode p-n Junction/AlGaN/GaN (PNJ) HEMT. IEEE Electron. Device Lett..

[33] Yu Z.J., Zhu Y., Gajadharsing J. Impact of Temperature-dependent Emission Time Constant of AlGaN/GaN HEMTs on DPD with Trapping Effects Compensation. Proceedings of the 2025 IEEE Topical Conference on RF/Microwave Power Amplifiers for Radio and Wireless Applications (PAWR).

[34] Chen J.T., Chen H.H., Cheng Y., Fang J.C., Wu Z., Li J.Q., Tang J.J., Zeng G.S., Chen K.J., Hua M.Y. (2025). Suppression of Drain-Bias-Induced VTH Instability in Schottky-Type p-GaN Gate HEMTs With Voltage Seatbelt. IEEE Trans. Electron. Devices.

